# Neural Correlates of Emotion Regulation in Substance Use

**DOI:** 10.1007/s40429-026-00782-8

**Published:** 2026-08-22

**Authors:** Sasha J. Hofman, Abigail M. Whitney, Tara M. Chaplin

**Affiliations:** Department of Psychology, George Mason University, Fairfax, VA 22030 USA

**Keywords:** Substance use, Substance use disorder, Emotion regulation, Functional magnetic resonance imaging (fMRI)

## Abstract

**Purpose of Review:**

Emotion regulation (ER) is a central process implicated in the development, maintenance, and escalation of substance use (SU) and substance use disorders (SUDs). Advances in functional magnetic resonance imaging (fMRI) have enabled characterization of neural systems underlying emotional processing, inhibitory control under emotional conditions, and deliberate ER in individuals engaging in SU. This review synthesizes recent neuroimaging findings related to ER and SU across three primary paradigms: emotional Go/No-Go tasks, cognitive reappraisal tasks, and implicit ER paradigms.

**Recent Findings:**

Across paradigms, SU is associated with altered engagement of frontolimbic, salience, and frontoparietal control networks, particularly during negative emotional contexts. Emotional Go/No-Go studies suggest that SU-related differences are most pronounced when inhibitory demands intersect with emotional salience. Cognitive reappraisal findings indicate disrupted recruitment and coordination of prefrontal–limbic systems during deliberate ER associated with SU, though results remain limited and mixed. Implicit ER paradigms further demonstrate context-dependent alterations in limbic reactivity and prefrontal connectivity associated with SU, with developmental and sex-specific patterns emerging in adolescent samples. Longitudinal findings suggest that some neural differences may precede SU initiation, whereas others may reflect consequences of chronic substance exposure.

**Summary:**

Collectively, this literature indicates that altered coordination between emotional salience detection and cognitive control systems represents a key neurobiological mechanism linking ER and SU. Future work integrating longitudinal, ecologically valid, and intervention-based designs will be critical for clarifying directionality and identifying modifiable neural targets to inform prevention and treatment efforts.

## Introduction

Emotion regulation (ER), broadly defined as the processes through which individuals monitor, evaluate, and modify emotional responses, has long been recognized as a central mechanism in the development and maintenance of substance use (SU) and substance use disorders (SUDs) [1, 2]. Individuals may use substances in an attempt to regulate emotions, including to reduce negative emotional states, alleviate craving, or enhance positive affective experiences [3–5]. Within this framework, the self-medication hypothesis emphasizes the use of substances to alleviate distressing internal states, proposing that SU is negatively reinforced by providing temporary relief from emotional discomfort [6]. Complementary models emphasize sensation-seeking and affective enhancement as the reasons for use, highlighting motivations to increase positive arousal or emotional intensity (i.e., up-regulation of positive emotional states) [4, 7]. Additionally, chronic and heavy SU may alter emotional arousal and neural systems underlying ER, leading to further declines in ER abilities among individuals with SUDs [8, 9]. Consistent with these perspectives, individuals with SUDs report greater ER difficulties than healthy controls, underscoring ER as a key process in problematic SU [10].

Recent advances in functional magnetic resonance imaging (fMRI) methodologies and task designs have supported growth in research examining the neural correlates of ER in individuals engaging in SU. This emerging literature has leveraged a range of experimental tasks to begin characterizing how neural responses to emotional stimuli and to ER demands are associated with SU across substances, developmental stages, and levels of use.

This review concentrates on recent fMRI studies examining neural markers of ER in youth and adults who engage in SU or who meet criteria for a SUD. The following fMRI task paradigms are reviewed: emotional Go/No-Go paradigms, which assess neural activation during *emotion processing* and during *inhibitory control under emotional conditions*; cognitive reappraisal paradigms, which assess neural activation during deliberate, top-down regulation of emotional responses (with participants instructed to reinterpret the meaning of emotional stimuli); and implicit ER paradigms, which assess neural activation during automatic, bottom-up processing of emotional responses to emotional stimuli (without explicit ER instructions), capturing how the brain regulates emotional information outside of conscious control. In addition, this review highlights new directions in the field, including emerging longitudinal and intervention-based studies, and identifies promising directions for future research.

### Emotional Go/No-Go Paradigms

Emotional Go/No-Go paradigms [11] are designed to assess how responses to emotionally salient stimuli influence emotion processing and inhibitory control. In these paradigms, *Go trials* require participants to execute a motor response (e.g., press a button) when a target stimulus appears, whereas *No-Go trials* require participants to withhold that response when a non-target stimulus appears. In emotional Go/No-Go paradigms, Go and No-Go trials are completed under emotional or neutral conditions (e.g., while viewing fearful or neutral facial expressions), which are presented either directly as task stimuli or as background images. Because emotional content and response demands vary across trials, these tasks allow researchers to isolate neural activation during different processes using contrasts. For example, contrasts may measure activation during *emotion processing* (e.g., Negative-Go > Neutral-Go) and during *inhibitory control under emotional conditions* (e.g., Negative-No-Go > Neutral No-Go) to capture ER processes, which may engage ER processes by requiring participants to regulate emotional responses in order to execute or withhold a response. Several recent studies using emotional Go/No-Go tasks have examined neural mechanisms of risk and impairment in SU, which we review below.

First, Droutman et al. [12] employed the emotional Go/No-Go paradigm examining adult women with SUDs in residential treatment. For each task trial, participants viewed an emotion word (happy, fear, or calm) followed by a set of emotionally congruent or incongruent faces. Participants were instructed to press a button when the facial expression was congruent with the preceding word (i.e., Go trial for target emotion) and to withhold responding when the expression was incongruent (i.e., No-Go trial for non-target emotion). To examine neural activation during *inhibitory control under negative emotional condition*s, (i.e., activation associated with inhibiting the natural avoidance response to fear), the authors contrasted activation during Go-Fear trials with Go-Happy trials and No-Go-Fear trials. Significant findings emerged using the Go-Fear > No-Go-Fear contrast, wherein women with SUDs showed higher activation to the Go-Fear > No-Go-Fear contrast in the inferior frontal gyrus (IFG), a region implicated in response inhibition and cognitive control, and the insula, a region implicated in salience detection. This finding could reflect that women with SUDs require higher engagement of control- and salience-related neural systems to down-regulate fear responses to complete the inhibition task, indicating high reactivity or challenges with ERhowever, this study did not include a healthy control group, so it is unclear if this pattern is specific to women with SUDs.

Another recent work examined SU dimensionally within a clinical population. Shi et al. [13] investigated adults with opioid use disorder (OUD) after detoxification and related neural responses during an emotional Go/No-Go task to indices of drug use severity. In this paradigm, participants were instructed to respond to positive emotional images (i.e., Go trials) and withhold responses to negative images (i.e., No-Go trials). To index what the authors termed emotional inhibitory control, a Negative No-Go > Positive-Go contrast was used. Using this contrast, greater drug use severity was associated with reduced activation in the frontoparietal control network, including the dorsolateral prefrontal cortex (dlPFC), which supports goal-directed control and working memory, in the posterior parietal cortex, implicated in attentional allocation, and in the dorsal attention network, which coordinates top-down attentional engagement. These findings indicate that higher levels of opioid use in individuals with OUD are linked to diminished recruitment of large-scale emotional regulatory and attentional systems during the inhibition of responses to negative emotional stimuli [13]. Of note, when examining emotional inhibitory control, using a Negative No-Go > Neutral No-Go contrast is more typical than Negative No-Go > Positive-Go. Consequently, emotional valence and motor demands were not separated in this study, so findings may reflect inhibitory processes, motor demands, or a combination of both.

An especially notable extension of the emotional Go/No-Go paradigm is provided by Dakhili et al. [14], who examined adults with methamphetamine use disorder (MUD) using a mixed task design that independently manipulated emotional valence, drug cue presence, and response inhibition. Participants responded to geometric shapes (i.e., triangles, squares, or diamonds; Go trials) and withheld responding to circles (i.e., No-Go trials), while background images were blank, neutral cues, negative emotional pictures, or drug-related cues. This design allowed the authors to test several contrasts separately. Significant between-group differences emerged in neural activation particularly during *inhibitory control under negative emotional conditions* (Negative No-Go > Neutral No-Go trials). Individuals with MUD showed greater activation than controls in temporal and parietal regions, regions that support integration of emotional and attentional information. This increased recruitment in those with MUD may reflect less efficient or compensatory processing during emotionally salient inhibition. Importantly, no group differences were observed during *emotion processing* (Combined Negative Go and No-Go > Combined Neutral Go and No-Go) or inhibitory control alone (No-Go > Go across all cue types). Instead, group differences emerged specifically when inhibitory demands occurred during negative emotional contexts. Such conditions may reflect real-world situations in which emotional states interfere with behavioral control and may require the downregulation of negative emotion in order to successfully inhibit responses.

Studies in adolescent and community samples reveal a somewhat different pattern. For example, Jones et al. [15] examined adolescents and young adults aged 11 to 21 years drawn from a community sample. SU was operationalized dimensionally as the number of lifetime use occasions rather than diagnostic status. Participants completed an emotional Go/No-Go task using happy, scared, and calm facial expressions. Contrasts reflected *emotion processing* (including Scared-Go > Calm-Go) and *inhibitory control under emotional conditions* (including Scared No-Go > Calm No-Go). Significant findings emerged only during *emotional processing* and not during *inhibitory control under emotional conditions*. During emotional processing of fearful stimuli (Scared-Go > Calm-Go), greater alcohol use was associated with *increased* activation in the middle cingulate cortex (MCC), implicated in conflict monitoring and action selection, while greater marijuana use was associated with *reduced* activation in the MCC and prefrontal regions, including the IFG, involved in response inhibition and cognitive control. These findings suggest that alcohol and marijuana use may be differentially associated with emotional cue processing during response execution, and that in this community sample of adolescents and young adults, substance-related differences were evident during emotion processing rather than during inhibition under emotional conditions.

Emotional Go/No-Go paradigms have also been used to identify emotion-related neural markers of SU risk prior to initiation, an approach that remains relatively uncommon in this literature. For instance, Cohen-Gilbert et al. [16] examined substance-naïve adolescents aged 13 to 14 years at baseline and followed them longitudinally for three years. Participants completed an emotional Go/No-Go task in which letters were superimposed on positive emotional, negative emotional, or neutral background images. Participants were instructed to respond to every letter (i.e., Go trials) and to withhold responding to the letter “X” (i.e., No-Go trials). *Inhibitory control under emotional conditions* was operationalized using a negative No-Go > neutral No-Go contrast. Reduced activation to the negative No-Go > neutral No-Go contrast in the left lateral frontoparietal network, involved in attention to salient information and coordination of action selection, predicted earlier SU initiation. Importantly, these neural differences were evident before any reported SU, highlighting their potential role as early markers of vulnerability rather than consequences of exposure.

Finally, Cohen-Gilbert et al. [17] used an emotional Go/No-Go task in a sample of college freshmen who engaged in a range of drinking behaviors, indexed by past three months’ binge episodes and total drinks consumed. Participants completed the emotional Go/No-Go task described in Cohen-Gilbert et al. [16], in which letters were presented over positive, negative, or neutral background images, and participants responded to every letter except “X.” *Inhibitory control under emotional conditions* was operationalized using negative No-Go > neutral No-Go and positive No-Go > neutral No-Go contrasts. Greater recent binge drinking was significantly associated with reduced activation to the negative No-Go > neutral No-Go contrast in the dlPFC, dorsomedial prefrontal cortex (dmPFC), and anterior cingulate cortex (ACC), regions strongly implicated in executive functioning and cognitive control. No significant associations were observed between binge drinking and neural activation for the positive No-Go > neutral No-Go contrast. These findings suggest that among young adults without alcohol use disorder (AUD), heavier binge drinking was associated with lower activation of inhibitory control circuitry under negative conditions, potentially reflecting an early vulnerability in ER processes that could confer risk for subsequent alcohol-related problems.

Taken together, studies using emotional Go/No-Go paradigms consistently implicate the prefrontal cortex, cingulate cortex, and distributed frontoparietal and attention networks during inhibitory control under emotional conditions and in emotion processing. During *inhibitory control under emotional conditions*, which may reflect regulation of emotion to achieve task goals, three studies found that adults with SUDs showed *heightened* activation in frontal emotional regulatory regions, attention networks, and tempo-parietal networks. One study of adults with OUD found lower activation in regulation and attention networks, but this study used an unusual contrast that did not parse out motor functioning. Overall, it appears that adults with SUDs may have less efficient neural engagement when regulating emotions during inhibitory processing. This may reflect that they require more effort to engage in regulation or have challenges in emotional arousal or regulation during cognitive tasks.

Notably, the three studies above using community samples of adolescents and younger adults (with relatively lower prior exposure to heavy SU) found either no association or that *lower* frontoparietal activation in emotional regulatory and attention networks during *inhibitory control under emotional conditions* associated with greater SU. This may suggest that, as adolescents and young adults, individuals may show lower activation and more efficient neural activation while regulating emotion during cognitive tasks and that this processing may become less efficient with the onset of SUDs and heavy substance exposure. Recent long-term longitudinal work, such as the Adolescent Brain Cognitive Development (ABCD) study [18], can test this possibility. It is also worth noting that the neural differences observed across these studies may not be specific to emotional contexts but could instead reflect a more global deficit in executive control, a transdiagnostic feature of SUD [2]. Future work comparing neural activation during neutral and emotional versions of the Go/No-Go task within the same SUD sample would be well-positioned to determine whether the effects reviewed here are emotion-specific or part of a broader impairment in inhibitory control (Table 1).

**Table 1:** Summary of Emotional Go/No-Go Studies

Study	Sample	Key Contrast	Main Findings
Droutman et al. [12]	Adult women with SUD in residential treatment	Go-Fear > No-Go-Fear	Higher IFG and insula activation in SUD group; no healthy control comparison
Shi et al. [13]	Adults with OUD post-detoxification	Negative No-Go > Positive-Go	Greater OUD severity associated with reduced dlPFC, posterior parietal, and dorsal attention network activation
Dakhili et al. [14]	Adults with MUD vs. healthy controls	Negative No-Go > Neutral No-Go; Combined Emotional > Neutral	Greater temporal/parietal activation in MUD during emotional inhibition; no group differences for emotion processing or neutral inhibition alone
Jones et al. [15]	Community adolescents and young adults (ages 11–21)	Scared-Go > Calm-Go; Scared No-Go > Calm No-Go	Greater alcohol use associated with increased MCC; greater marijuana use associated with reduced MCC and IFG during emotion processing; no group differences during emotional inhibitory control
Cohen-Gilbert et al. [16]	Substance-naïve adolescents (ages 13–14), 3-year longitudinal follow-up	Negative No-Go > Neutral No-Go	Reduced left frontoparietal network activation predicted earlier SU initiation
Cohen-Gilbert et al. [17]	College freshmen with varying drinking behaviors	Negative No-Go > Neutral No-Go	Greater binge drinking associated with reduced dlPFC, dmPFC, and ACC activation during emotional inhibitory control

### Cognitive Reappraisal Paradigms

Cognitive reappraisal paradigms are designed to examine deliberate ER by asking participants to reinterpret the meaning of negative stimuli in order to downregulate emotional responses. A commonly used paradigm is the Cognitive Reappraisal Task [19], in which participants are shown negative emotional or neutral image stimuli. Prior to scanning, participants are taught cognitive reappraisal strategies and are instructed during the task to either: Observe neutral stimuli, Maintain their emotional response to negative stimuli, or Regulate their emotional response to negative stimuli. This design allows for a clear distinction between neural activation during *emotion processing*, indexed by relative brain activation during Maintain > Observe conditions, and during *emotion regulation*, indexed by relative activation during Regulate > Maintain conditions. In contrast to emotion-inhibition paradigms, this task places explicit demands on top-down control processes and provides a direct probe of deliberate ER capacity.

Overall, research using cognitive reappraisal paradigms in the context of SU is limited. Two prior reviews in 2016 and 2017 of ER in SU noted that, at the time of publication, only one fMRI study had examined cognitive reappraisal in individuals engaging in SU [2, 20]. That study, by Albein-Urios et al. [21], found that abstinent adults with cocaine dependence showed greater activation than controls in prefrontal regions, which are involved in regulation, during *emotion processing* (Maintain > Observe) and showed reduced activation in prefrontal, insular, posterior cingulate, and sensory regions, involved in regulation/control and salience detection, along with reduced IFG–amygdala coupling, regions involved in cognitive control and emotion generation, during *emotion regulation* (Regulate > Maintain) [21]. Two studies published since 2017 have expanded this line of work, which are reviewed below.

First, Pico-Perez et al. [22] examined adults with cocaine use disorder (CUD) and healthy controls using the same Cognitive Reappraisal Task employed in Albein-Urios et al. [21], as well as the same cocaine-dependent sample, but applied a large-scale network analytic approach rather than focusing primarily on regional activation. During *emotion processing* (Maintain > Observe), individuals with CUD showed reduced activation in limbic networks, including the amygdala and hippocampus, which support emotion generation and memory-based appraisal, alongside increased engagement of ventral frontostriatal circuitry, encompassing the ventral striatum and medial prefrontal cortex, regions involved in reward valuation and motivational salience. During *emotion regulation* (Regulate > Maintain), reduced engagement of attention-related networks was observed in the cocaine-dependent group relative to controls, suggesting diminished allocation of cognitive resources during deliberate ER. When considered alongside the regional findings reported by Albein-Urios et al. [21], these results point to broadly similar neural circuity involvement in emotion processing and regulation.

Not all studies examining deliberate reappraisal report clear neural differences between individuals engaging in SU and controls. Hiebler-Ragger et al. [23] examined adults with polydrug use disorder receiving inpatient treatment and controls using the Reappraisal Generation Task [24]. Rather than instructing participants to apply predefined strategies, this task required participants to spontaneously generate and verbalize cognitive reinterpretations of aversive situations during scanning. During reappraisal, both polydrug use disorder participants and control participants showed engagement of lateral prefrontal regions, including the superior and middle frontal gyri, which are implicated in executive control processes, and no significant group differences in neural activation were observed.

Across these cognitive reappraisal studies, several converging themes emerge. Of the two studies reviewed, Pico-Perez et al. [22] found that adults with CUD showed altered limbic and prefrontal recruitment during both emotion processing and regulation relative to controls, consistent with patterns reported in the prior study by Albein-Urios et al. [21]. In contrast, Hiebler-Ragger et al. [23], which examined polydrug users, did not find group differences during reappraisal. In these studies, participants with CUD generally showed heightened neural activation in prefrontal and striatal regions during *emotion processing* relative to controls (though findings were mixed for limbic activation, with one study finding higher and one finding lower activation in limbic regions). This pattern may suggest that SU is associated with a heightened emotional reactivity, which may lead to increased SU to down-regulate negative emotions, consistent with the self-medication hypothesis. Regarding neural activation during *emotion regulation*, the Albein-Urios and Pico-Perez [21, 22] studies both found lower activation (i.e., lower engagement) in emotional regulatory, attention, and salience regions during ER in participants with CUD, which may lead to challenges in regulating emotions. In contrast, the study of polydrug users did not find group differences, possibly due to the absence of explicit regulation instructions in this task. Overall, there are few studies examining neural activation during explicit ER for individuals engaging in SU, and none in adolescence or community samples. Thus, future research should examine these processes in more developmentally and clinically diverse populations (Table 2).

**Table 2:** Summary of Cognitive Reappraisal Studies

Study	Sample	Key Contrast	Main Findings
Albein-Urios et al. [21]	Abstinent adults with cocaine dependence vs. healthy controls	Maintain > Observe; Regulate > Maintain	Higher prefrontal activation during emotion processing; reduced prefrontal, insular, PCC activation, and IFG–amygdala coupling during regulation in CUD
Picó-Pérez et al. [22]	Adults with CUD vs. healthy controls (network-level analysis)	Maintain > Observe; Regulate > Maintain	Reduced limbic and increased frontostriatal activation during emotion processing; reduced attention network engagement during regulation in CUD
Hiebler-Ragger et al. [23]	Adults with polydrug use disorder in inpatient treatment vs. controls	Reappraisal generation vs. baseline	No significant group differences in prefrontal or other regional activation during reappraisal

### Implicit ER Paradigms

Implicit ER paradigms assess how emotional information is managed when regulation is not explicitly instructed. Rather than asking participants to deliberately modulate their emotional responses, these tasks infer ER processes from neural engagement during emotional interference or passive viewing. As such, they are well suited to probing more automatic or spontaneous forms of ER. Prior work has found that in tasks that did not explicitly instruct participants to regulate emotion, individuals engaging in SU frequently showed altered limbic reactivity to negative emotional stimuli and disrupted recruitment or coordination of prefrontal emotional regulatory systems (for review, see Wilcox et al. [2]). These patterns were interpreted as reflecting inefficiencies in more automatic or implicit forms of ER. Below we review recent studies, which extend prior work by examining how these implicit ER-related circuits operate across developmental periods, environmental risk contexts, and longitudinal trajectories of SU.

First, Zhornitsky et al. [25] examined emotional processing using a passive viewing paradigm in adults with cocaine use disorder (CUD) and controls. Participants viewed negative and neutral images. Compared to controls, individuals with CUD showed greater activation to negative images (versus neutral) in limbic regions, including the hippocampus, which supports emotional memory and contextual encoding, as well as in parietal regions implicated in attentional processing. This pattern suggests heightened emotional and attentional engagement in response to negative emotional stimuli in CUD, even in the absence of explicit ER demands.

Additional insight into implicit regulatory dynamics is provided by Hammond et al. [26], who examined adults with AUD and controls using an emotional face-matching task. Participants matched angry or fearful faces (or geometric shapes), and neural responses were modeled using the contrast of emotional face-matching > shape-matching. Importantly, groups did not differ in contrast-elicited regional activation. However, dynamic causal modeling revealed significant differences in effective (directional) connectivity during emotional face processing. Relative to controls, individuals with AUD exhibited widespread alterations in effective connectivity across prefrontal, limbic, and perceptual regions during matching of angry and fearful faces relative to shape-matching, including reduced top-down connectivity from ventromedial and ventrolateral prefrontal areas and altered bidirectional connectivity among PFC, amygdala, fusiform gyrus, and hypothalamic regions. Several of these connectivity pathways were associated with cumulative alcohol exposure, indicating that alcohol involvement was related to altered coordination between control and emotion-related circuitry. These findings suggest that during implicit emotional face processing, ER differences in AUD may be more evident in circuit-level interactions than in regional activation alone.

Next, Perini et al. [27] used an emotional conflict task to examine neural mechanisms of resilience to SUD following childhood maltreatment. In this study, emotional conflict involved identifying emotion in an emotional face picture while ignoring an emotionally-incongruent word superimposed on the face, which requires the engagement of implicit ER processes in order to inhibit emotional responses. Contrasts were constructed based on whether the trial was congruent (e.g., “fear” superimposed on fearful face image) or incongruent (e.g., “fear” superimposed on happy face image). Adults were classified based on childhood maltreatment history and lifetime SUD status, allowing for direct comparison between maltreated individuals who developed an SUD (SUD+maltreatment group) and did not develop an SUD (control+maltreatment group). During emotional conflict processing (i.e., during incongruent trials), the SUD+maltreatment group showed lower activation in regions implicated in implicit ER and salience processing, including the medial prefrontal cortex, ACC, and insula, relative to the control+maltreatment group. Additionally, non-maltreated controls generally showed intermediate or normative activation patterns. These results suggest that in the context of childhood maltreatment, individuals who developed SUD showed reduced recruitment of emotional regulatory and salience-related regions during emotional interference, whereas maltreated individuals without SUD showed greater engagement of these systems. This pattern is consistent with a resilience model, in which preserved or enhanced engagement of regulatory circuitry reflects more adaptive implicit ER following early adversity.

Consistent with the adult findings above, several recent studies have extended implicit ER paradigms to adolescent samples. Leiker et al. [28] examined adolescents with behavioral, emotional, and SU problems aged 14 to 18 years using a face-processing task in which participants identified the gender of faces displaying neutral, happy, or fearful expressions. Because participants were not instructed to regulate their emotional responses, this task captures neural responses to emotional stimuli under incidental processing demands, providing an index of implicit ER. Using a Fear > Neutral contrast, greater alcohol use severity was associated with reduced activation in the left inferior parietal lobule, which is implicated in attentional allocation and integration of emotionally salient information. Cannabis use severity did not show a similar association. These findings suggest that greater alcohol use severity in adolescence may be linked to blunted neural activation to negative emotional stimuli within attentional networks, reflecting altered implicit regulation of emotional salience, though findings should be interpreted alongside substantial psychiatric comorbidity with greater SU severity in this sample.

In our lab, we have also examined implicit ER in early adolescence. In a community sample of youth aged 12 to 14 years, Chaplin et al. [28] found that heightened left anterior insula activation during Negative > Neutral image viewing was associated with greater likelihood of SU in girls but not boys. The anterior insula plays a central role in salience detection and interoceptive awareness, suggesting that heightened sensitivity to negative emotional cues may confer vulnerability to SU among girls. Extending this work longitudinally in a larger cohort of community youth oversampled for risk via maladaptive parenting aged 12 to 14 years, Chaplin et al. [29] found that neural activation to Negative > Neutral images did not predict initiation of SU. However, among adolescents who did initiate use, heightened activation to Negative > Neutral images in the right amygdala, central to threat detection and emotional arousal, predicted steeper growth in SU frequency over a three-year longitudinal follow-up period. This effect was specific to girls, with no significant associations observed for boys. These findings suggest that for girls, elevated negative emotional reactivity in early adolescence may contribute to escalation of SU. Together, these three adolescent study findings underscore that variability in neural activation during incidental emotional processing, even in the absence of instructed regulation, may reflect differences in implicit ER that confer vulnerability to SU, with sex-specific patterns emerging during escalation.

Overall, studies using implicit ER tasks suggest that SU–related differences most consistently involve limbic, salience, and frontoparietal systems, though the direction of effects varies by developmental stage and task demands. In passive viewing paradigms using Negative > Neutral contrasts, adults with CUD and adolescents at risk for escalation showed heightened activation in limbic and salience-related regions, including the amygdala, hippocampus, and insula, pointing toward increased negative emotional reactivity in the absence of explicit regulation [25, 29, 30]. In contrast, one adolescent study with a clinical sample of adolescents found reduced activation in the inferior parietal lobule, a region supporting attentional allocation to salient stimuli, suggesting blunted modulation of negative cues in youth with greater alcohol symptoms [28]. When tasks required management of emotional interference or matching demands, alterations were more evident in prefrontal and cingulate systems implicated in implicit emotional regulatory control. Maltreated individuals who developed SUD showed reduced activation in medial prefrontal, anterior cingulate, and insular regions during emotional conflict relative to resilient maltreated individuals [27]. Similarly, adults with AUD demonstrated altered effective connectivity among prefrontal and limbic regions during emotional face processing [26]. Together, these findings suggest that implicit ER in SU is characterized by dysregulation within overlapping salience and control networks and is context-dependent, manifesting either as heightened limbic reactivity or reduced recruitment and coordination of prefrontal–cingulate systems (Table 3).

**Table 3:** Summary of Implicit Emotion Regulation Studies

Study	Sample	Task/Contrast	Main Findings
Zhornitsky et al. [25]	Adults with CUD vs. healthy controls	Passive viewing: Negative > Neutral images	Greater hippocampal and parietal activation in CUD; heightened emotional and attentional engagement to negative stimuli
Hammond et al. [26]	Adults with AUD vs. healthy controls	Emotional face-matching: Emotional faces > Geometric shapes	No regional activation differences; widespread alterations in effective connectivity among prefrontal, limbic, and perceptual regions in AUD
Perini et al. [27]	Adults with childhood maltreatment, with vs. without lifetime SUD	Emotional conflict task: Incongruent > Congruent trials	SUD+maltreatment group showed reduced mPFC, ACC, and insula activation relative to maltreated individuals without SUD
Leiker et al. [28]	Adolescents with behavioral and SU problems (ages 14–18)	Gender identification of emotional faces: Fear > Neutral	Greater alcohol severity associated with reduced left inferior parietal lobule activation; no association for cannabis severity
Chaplin et al. [29]	Community youth (ages 12–14)	Passive viewing: Negative > Neutral images	Heightened left anterior insula activation associated with greater SU likelihood in girls only
Chaplin et al. [30]	Community youth (ages 12–14), 3-year longitudinal follow-up	Passive viewing: Negative > Neutral images	Heightened right amygdala activation predicted steeper SU escalation in girls who initiated use; no significant effect for boys

## Conclusions

### Emerging Findings and Broader Implications

Across paradigms, several patterns emerge regarding neural activation during emotional processing, inhibitory control under emotional conditions, and ER in individuals engaging in SU. During emotional processing contrasts (e.g., Maintain > Observe; Negative > Neutral; Fear-Go > Calm-Go), many studies report heightened activation in limbic and salience-related regions, including the amygdala, insula, hippocampus, and cingulate cortex, particularly in adults with CUD and in adolescents at risk for SU escalation [25, 29–31]. At the same time, other studies show blunted recruitment in frontoparietal and attentional control regions during both emotional processing and inhibitory control under emotional conditions, particularly in youth or adults with more severe SU [13, 16, 17, 28]. Findings from emotional Go/No-Go paradigms further suggest that SU-related differences may be most evident when inhibitory demands occur in negative contexts, rather than during inhibition or emotion processing alone [14].

During explicit ER contrasts (e.g., Regulate > Maintain), prefrontal and parietal control systems are consistently implicated. Some studies report increased recruitment of dorsolateral prefrontal and attentional regions, potentially reflecting greater emotional regulatory effort [31], whereasothers find reduced activation or weakened prefrontal-limbic functional coupling, suggesting less efficient coordination of control systems during attempts to downregulate negative affect [21, 22, 26].

Collectively, this work indicates that SU is associated with altered engagement of neural systems supporting emotional salience detection and goal-directed control, particularly when negative emotion and behavioral demands intersect. These alterations may increase reliance on substances as a means of modulating distress or arousal; however, it is also possible that repeated heavy SU contributes to progressive changes in frontolimbic and control networks over time. Longitudinal and intervention-focused research will be critical for disentangling directionality and clarifying how these neural patterns relate to risk, maintenance, and recovery.

### Future Directions

This review suggests several interesting future directions. First, recent work on the neural correlates of ER in SU reflect a growing interest in moving beyond static group differences toward questions of change, vulnerability, and clinical relevance. Although much of the literature remains cross-sectional, findings from intervention-focused work suggest that ER-related neural systems may be modifiable. For example, Froeliger et al. [32] found that changes in neural engagement during ER following a mindfulness-based intervention were associated with reductions in nicotine use, linking within-person neural change to meaningful behavioral outcomes. Together with studies showing altered prefrontal, salience, and limbic engagement across Go/No-Go, reappraisal, and implicit paradigms, this work raises the possibility that these neural markers are not only correlates of SU but potential targets that can be modulated by intervention.

Further, longitudinal and risk-focused designs are beginning to clarify whether ER-related neural differences precede or follow SU. Reviewed findings collectively suggest that ER-related neural function may operate as both a marker of vulnerability and a consequence of SU exposure, underscoring the need for additional longitudinal studies that track neural and behavioral change over time. With respect to implicit ER, the relationship between neural reactivity and SU appears to differ meaningfully across development and sex [25, 28–30]; as such, future longitudinal work should examine developmental and sex-specific pathways to clarify how these associations emerge and change over time. More broadly, a central theme across implicit ER studies is that effects were highly context-dependent, with passive viewing paradigms more consistently revealing heightened limbic and salience-related reactivity in adults with CUD and adolescents at risk for escalation [25, 29, 30], whereas paradigms requiring active management of emotional interference more commonly showed reduced prefrontal and anterior cingulate recruitment [26, 27]. Future research should examine what specific task and contextual demands drive these divergent patterns, which would help identify the particular emotional or behavioral contexts in which implicit ER difficulties confer the greatest SU risk.

Next, methodological advances highlight the importance of studying ER in contexts that more closely reflect real-world emotional challenges. Emerging evidence suggests that SU-related differences are most apparent when emotional salience and cognitive control demands intersect [14], and that alterations may be better characterized at the level of circuit coordination rather than isolated regional activation [26]. Moving forward, research should prioritize more ecologically valid paradigms that capture emotionally meaningful stimuli, such as personalized negative emotional images or videos drawn from participants’ daily lives [e.g., 9, 33], an approach we are currently implementing in our lab. For example, we conducted a study in which adolescents viewed videos of their own parents expressing positive and negative emotion towards them, drawn from a laboratory task conducted two weeks prior [33]. In this study, we found that higher nucleus accumbens activation to videos of adolescents’ own parents showing positive emotions was linked at a trend level with greater adolescent SU. Such approaches may better model the types of emotionally charged situations in which SU risk unfolds.

Future studies may also integrate fMRI with ecological momentary assessment (EMA) to link neural markers of ER with real-time fluctuations in emotion and SU in naturalistic settings. This multimethod approach allows researchers to test whether neural indices of emotional reactivity or regulation moderate momentary associations between emotions and SU in daily life. For example, preliminary work from our lab found that adolescents’ momentary negative emotional experiences in daily life predicted their momentary SU and externalizing symptoms, particularly among those showing blunted amygdala activation to negative emotional images during fMRI [34].

Overall, this review underscores that alterations in neural systems supporting emotional processing, salience detection, and cognitive control, all of which are related to ER, are closely intertwined with SU across development. This work supports the development of neuroscience-informed interventions that focus on emotional processing and regulation to prevent and reduce SU.

## Key References

- Jones SA, Del Giacco AC, Barnes SJ, Nagel BJ. Adolescent substance use is associated with altered brain response during processing of negative emotional stimuli. J Stud Alcohol Drugs. 2023;84(2):257–266. 10.15288/jsad.22-00143.
  - **○ **Found that during emotional processing in scared versus calm conditions, greater alcohol use, but not marijuana use, was associated with activation in the middle cingulate cortex.
- Cohen-Gilbert JE, Sneider JT, Oot EN, Seraikas AM, Schuttenberg EM, Harris SK, et al. Risk for adolescent substance use initiation: associations with large-scale brain network recruitment during emotional inhibitory control. Beh Sci (Basel). 2025;15(10):1407. 10.3390/bs15101407.
  - **○ **Identified potential neural markers for early substance use in adolescents.
- Hammond CJ, Ma L, Bjork JM, Gerard Moeller F, Arias AJ. Altered effective connectivity of emotion perception and regulation networks during an emotional face perception task in adults with alcohol use disorder. Brain Struct Funct. 2025;230(7):136. 10.1007/s00429-025-02992-8.
  - **○ **Identified alterations in effective connectivity in individuals with substance use disorder during an emotional face-matching task.
- Perini I, Mayo LM, Capusan AJ, Paul ER, Yngve A, Kampe R, et al. Resilience to substance use disorder following childhood maltreatment: association with peripheral biomarkers of endocannabinoid function and neural indices of emotion regulation. Mol Psychiatry. 2023;28(6):2563–71. 10.1038/s41380-023-02033-y.
  - **○ **Found that individuals who did not develop substance use disorder following childhood maltreatment presented higher engagement in emotion regulation and salience regions compared to those that did develop substance use disorder during an emotional interference task.
- Chaplin TM, Curby TW, Gonçalves SF, Kisner MA, Niehaus CE, Thompson JC. Sex differences in emotion- and reward-related neural responses predicting increases in substance use in adolescence. Behav Brain Res. 2023;450:114499. 10.1016/j.bbr.2023.114499.
  - **○ **Found that among adolescent girls with initiated substance use, heightened activation in the right amygdala to negative emotional stimuli longitudinally predicted growth in substance use frequency.

## Data Availability

No datasets were generated or analysed during the current study.
